# Comparison of Tc-99m maraciclatide and Tc-99m sestamibi molecular breast imaging in patients with suspected breast cancer

**DOI:** 10.1186/s13550-017-0255-6

**Published:** 2017-01-14

**Authors:** Michael K. O’Connor, Melissa M. B. Morrow, Katie N. Hunt, Judy C. Boughey, Dietlind L. Wahner-Roedler, Amy Lynn Conners, Deborah J. Rhodes, Carrie B. Hruska

**Affiliations:** 1 Department of Radiology, Mayo Clinic, Charlton 1-225, 200 First Street SW, Rochester, 55905 MN USA; 2 Health Sciences Research and the Kern Center for the Science of Healthcare Delivery, Mayo Clinic, 200 First Street SW, Rochester, 55905 MN USA; 3 Department of Surgery, Mayo Clinic, 200 First Street SW, Rochester, 55905 MN USA; 4 Department of Internal Medicine, Mayo Clinic, 200 First Street SW, Rochester, 55905 MN USA

**Keywords:** Tc-99m sestamibi, Tc-99m maraciclatide, Tc-99m NC100692, Breast cancer, Molecular breast imaging

## Abstract

**Background:**

Molecular breast imaging (MBI) performed with ^99m^Tc sestamibi has been shown to be a valuable technique for the detection of breast cancer. Alternative radiotracers such as ^99m^Tc maraciclatide may offer improved uptake in breast lesions. The purpose of this study was to compare relative performance of ^99m^Tc sestamibi and ^99m^Tc maraciclatide in patients with suspected breast cancer, using a high-resolution dedicated gamma camera for MBI. Women with breast lesions suspicious for malignancy were recruited to undergo two MBI examinations—one with ^99m^Tc sestamibi and one with ^99m^Tc maraciclatide. A radiologist interpreted MBI studies in a randomized, blinded fashion to assign an assessment score (1–5) and measured lesion size. Lesion-to-background (L/B) ratio was measured with region-of-interest analysis.

**Results:**

Among 39 analyzable patients, 21 malignant tumors were identified in 21 patients. Eighteen of 21 tumors (86%) were seen on ^99m^Tc sestamibi MBI and 19 of 21 (90%) were seen on ^99m^Tc maraciclatide MBI (*p* = 1). Tumor extent measured with both radiopharmaceuticals correlated strongly with pathologic size (^99m^Tc sestamibi, *r* = 0.84; ^99m^Tc maraciclatide, *r* = 0.81). The L/B ratio in detected breast cancers was similar for the two radiopharmaceuticals: 1.55 ± 0.36 (mean ± S.D.) for ^99m^Tc sestamibi and 1.62 ± 0.37 (mean ± S.D.) for ^99m^Tc maraciclatide (*p* = 0.53). No correlation was found between the L/B ratio and molecular subtype for ^99m^Tc sestamibi (*r*_*s*_ = 0.12, *p* = 0.63) or ^99m^Tc maraciclatide (*r*_*s*_ = −0.12, *p* = 0.64). Of 20 benign lesions, 10 (50%) were seen on ^99m^Tc sestamibi and 9 of 20 (45%) were seen on ^99m^Tc maraciclatide images (*p* = 0.1). The average L/B ratio for benign lesions was 1.34 ±0.40 (mean ±S.D.) for ^99m^Tc sestamibi and 1.41 ±0.52 (mean ±S.D.) for ^99m^Tc maraciclatide (*p* = 0.75). Overall diagnostic performance was similar for both radiopharmaceuticals. AUC from ROC analysis was 0.83 for ^99m^Tc sestamibi and 0.87 for ^99m^Tc maraciclatide (*p* = 0.64).

**Conclusions:**

^99m^Tc maraciclatide offered comparable lesion uptake to ^99m^Tc sestamibi, in both malignant and benign lesions. There was good correlation between lesion extent and uptake measured from both radiopharmaceuticals. ^99m^Tc maraciclatide offered a marginal (but not significant) improvement in sensitivity over ^99m^Tc sestamibi. Our findings did not support an association between the uptake of either radiopharmaceutical and tumor molecular subtype.

**Trial registration:**

ClinicalTrials.gov, NCT00888589

## Background

Molecular breast imaging (MBI) is a nuclear medicine technique that utilizes a specialized gamma camera system optimized for breast imaging. Because MBI relies on the preferential uptake of a radiopharmaceutical in metabolically active cells to distinguish breast abnormalities from normal parenchyma, the sensitivity of this technique is not affected by mammographic breast density and has been shown to improve detection of mammographically occult cancers. In recent studies, supplemental screening with MBI has been shown to detect an additional 7.5 to 16.5 cancers per 1000 women screened [1].

To date, most studies of MBI have utilized the radiopharmaceutical ^99m^Tc sestamibi, with only limited reports on the use of alternative radiopharmaceuticals for breast imaging [2–6]. Tc-99m sestamibi is primarily used as a perfusion agent for cardiac imaging, and was serendipitously discovered to also accumulate in breast cancer. The biodistribution of Tc-99m sestamibi is such that the relative uptake of Tc-99m sestamibi in breast cells is a factor of 20 lower than in myocardial cells [7], and hence only a small proportion of administered activity is sequestered in breast tissue. Despite this low uptake, MBI with Tc-99m sestamibi has been shown to be highly sensitive for the detection of breast cancers [1]. However some tumors are not well-visualized on MBI. In a series of 286 patients with 390 tumor foci, 49 (12%) were occult on MBI; most of the lesions that were occult on MBI with Tc-99m sestamibi were either small (5 mm or less) and potentially below the resolving power of the camera system or located outside the field of view; however, in 17 of 49 (35%), the reason for the absence of Tc-99m sestamibi uptake in the lesion was unknown [8].

An ideal alternative radiopharmaceutical to Tc-99m sestamibi would have a more favorable biodistribution to breast tissue and offer better visualization of breast tumors through higher uptake in breast malignancies relative to the amount of uptake in normal breast tissue. A radiopharmaceutical of particular interest is the imaging agent ^99m^Tc-NC100692 (also known as Maraciclatide, GE Healthcare), which is a synthetic cyclic peptide with high affinity for specific integrins, such as αvβ3, that are upregulated during angiogenesis [9]. Angiogenesis is critical for the growth of solid tumors as tumor growth beyond a volume of 1–2 mm^3^ requires independent vasculature [10, 11]. An indirect approach to imaging angiogenesis has focused on radiotracers targeting the integrin αvβ3 receptors which are significantly upregulated in endothelial cells during angiogenesis and are known to be expressed in breast cancer [12].

The αvβ3 integrin is a membrane-spanning protein that is expressed preferentially on proliferating endothelial cells associated with neovascularization but is absent in quiescent blood vessels [13, 14]. The binding of ^99m^Tc maraciclatide has been confirmed to be localized to endothelial cells in the regions of angiogenesis [15], and may provide a promising alternative radiopharmaceutical to ^99m^Tc sestamibi. Bach-Gansmo et al. first reported on the use of a ^99m^Tc-labeled angiogenesis agent (^99m^Tc NC100692) for the detection of breast cancers using MBI [16, 17].

The purpose of this study was to compare relative performance of ^99m^Tc sestamibi and ^99m^Tc maraciclatide in patients with suspected breast cancer, using a high-resolution dedicated gamma camera for MBI.

## Methods

### Study population

This study was performed under an IRB-approved, Health Insurance Portability and Accountability Act-compliant research protocol, and written informed consent was obtained from all participants. As ^99m^Tc maraciclatide is a not an FDA-approved radiopharmaceutical, this study was performed under an investigator IND cross-referred to the IND held by the radiopharmaceutical manufacturer, GE Healthcare Life Sciences (Pittsburgh, PA).

Female patients with known or suspected breast cancer were offered participation in the study. Eligible patients included those with at least one breast lesion identified by clinical findings, mammography, targeted ultrasound, or magnetic resonance imaging that was considered suspicious or highly suggestive of malignancy on the American College of Radiology Breast Imaging Reporting and Data System (BI-RADS) scale [18] and scheduled for biopsy. MBI examinations were performed prior to breast biopsy when possible (*N* = 32). Patients were also eligible to have MBI performed after breast biopsy if the lesion was pathologically proven as malignant and estimated to be at least 1.5 cm in maximum diameter (*N* = 7). This requirement on lesion size was to avoid recruitment of patients in whom a significant portion of the lesion would likely be removed at the time of biopsy.

### Molecular breast imaging examinations

Patients underwent two MBI examinations—one with ^99m^Tc sestamibi and one with ^99m^Tc maraciclatide. MBI was performed on a dual-head system that comprised two compact cadmium zinc telluride detectors with 1.6 × 1.6 mm pixels (LumaGem system, Gamma Medica, Salem, NH) and was equipped with high sensitivity registered tungsten collimators [19]. An energy acceptance window of 110–154 keV was used [20, 21].

When possible, patients were scheduled for ^99m^Tc sestamibi and ^99m^Tc maraciclatide MBI examinations on separate days (2-day protocol; *N* = 20). If the patient schedule did not permit a 2-day protocol, then a 1-day protocol was followed (*N* = 19), as described below. For logistical reasons related to preparation and quality control of the ^99m^Tc maraciclatide and to the wait time after injection of the ^99m^Tc maraciclatide, the order of the tests was not randomized. For all patients, MBI with ^99m^Tc sestamibi was performed first, followed by MBI with ^99m^Tc maraciclatide. Between 24–72 h after injection of the ^99m^Tc maraciclatide, patients were contacted to determine if any adverse events (AEs) occurred.

#### 2-day protocol

Patients received an intravenous injection of 300 MBq (8 mCi) ^99m^Tc sestamibi for the first MBI scan on day 1 and 300 MBq (8 mCi) ^99m^Tc maraciclatide for the second MBI scan on day 2, performed at least 16 h after the day 1 examination. Injections were given in the contralateral arm to the breast with the suspected lesion and residual syringe activity was measured after injection. The times at which the injections were performed, imaging was commenced, and dose activities were measured, were recorded to permit accurate correction for decay of the ^99m^Tc and computation of administered activities. Imaging commenced approximately 5 min post-injection with ^99m^Tc sestamibi and approximately 45 min post-injection with ^99m^Tc maraciclatide. The difference in wait time before imaging with ^99m^Tc sestamibi and ^99m^Tc maraciclatide was to allow for known differences in rate of uptake of these radiopharmaceuticals in breast tumors [16]. Bilateral cranio-caudal (CC) and medio-lateral oblique (MLO) analogous views were obtained under light compression for 10 min per view for a total imaging time of 40 min. The compressed breast thickness for each view acquired during the ^99m^Tc sestamibi MBI examination was recorded and replicated at the ^99m^Tc maraciclatide MBI examination to assist with consistent positioning of the breast between studies.

#### 1-day protocol

The 1-day protocol procedure was similar to that described above, except that after completion of the first MBI scan, performed with 300-MBq (8 mCi) injection of ^99m^Tc sestamibi, patients were immediately injected with 740 MBq (20 mCi) ^99m^Tc maraciclatide for the second MBI scan. Approximately 45 min post-injection of the ^99m^Tc maraciclatide, images were acquired as described above.

The rationale for the higher administered dose of ^99m^Tc maraciclatide for the 1-day protocol was to minimize the contribution of ^99m^Tc sestamibi to the second scan. The time interval from injection of the ^99m^Tc sestamibi to acquisition of the ^99m^Tc maraciclatide images was approximately 90 min for each patient (45 min ^99m^Tc sestamibi injection and imaging, 45 min wait after injection of ^99m^Tc maraciclatide) allowing for physical decay of the ^99m^Tc sestamibi to at least 250 MBq, and physical decay of the ^99m^Tc maraciclatide to 680 MBq. No adjustment was made for any biological washout. Therefore, the relative contribution of counts from the ^99m^Tc maraciclatide and ^99m^Tc sestamibi injections in the second MBI scan was anticipated to be approximately 2.7:1 (~680 MBq ^99m^Tc maraciclatide/~250 MBq ^99m^Tc sestamibi).

### Histopathology

Histopathologic classification was by the most severe of findings from surgical excision or by core needle or vacuum-assisted biopsy. Tumor size was obtained from the maximum tumor extent reported on pathology. Molecular subtype of malignant lesions was characterized as luminal (ER+ and HER2−), HER2-enriched (HER2+/ER−/PR−), or triple negative (ER−/PR−/HER2−).

### Image analysis

Image manipulation and region of interest (ROI) analysis were performed on the ^99m^Tc maraciclatide and ^99m^Tc sestamibi MBI images using an Xeleris workstation (GE Healthcare, Milwaukee, WI).

Radiopharmaceutical uptake in breast lesions was measured as a lesion-to-background (L/B) ratio, performed as follows. A 3 × 3 median filter was first applied to all images to minimize noise. An ROI was manually drawn to encompass the area of focal uptake corresponding to the lesion using the upper or lower detector CC or MLO view that best visualized the lesion. This lesion ROI was copied to the complementary upper or lower detector image. The maximum count in each lesion ROI was obtained and a geometric mean (GM) of the two counts was calculated. Background activity was measured from a second circumferential ROI that was drawn over a region of normal tissue surrounding the lesion. This background ROI was again copied to the corresponding area of normal tissue on the upper or lower detector image. The average count in each background ROI was obtained and a geometric mean value was calculated. L/B ratio was defined as

$$ \mathrm{L}/\mathrm{B}\ \mathrm{ratio} = \mathrm{G}\mathrm{M}\ \left(\mathrm{maximum}\ \mathrm{counts}\ \mathrm{in}\ \mathrm{lesion}\right)\ /\ \mathrm{G}\mathrm{M}\ \left(\mathrm{average}\ \mathrm{counts}\ \mathrm{in}\ \mathrm{background}\right) $$

For the 1-day protocol studies, a cross-talk correction was applied as follows. Count measurements from the lesion and background ROIs from the ^99m^Tc sestamibi images were decay corrected for the time interval between the ^99m^Tc maraciclatide and ^99m^Tc sestamibi acquisitions and reduced further by a factor of 0.94 (to adjust for biological washout of the ^99m^Tc sestamibi from breast tissue) [22]. These counts were then subtracted from counts in the ^99m^Tc maraciclatide images. No cross-talk correction was necessary for the 2-day protocol.

### MBI interpretation

A breast imaging fellowship-trained radiologist with 4 years of experience interpreting MBI (KNH) performed two independent reading sessions, separated by 4 weeks, of the MBI studies. Each session comprised a random order of ^99m^Tc sestamibi and ^99m^Tc maraciclatide studies; only one study appeared for each patient per session. The radiologist was blinded to the radiopharmaceutical, other imaging findings, and all clinical information including pathology findings. MBI studies were interpreted according to a validated lexicon for gamma imaging of the breast [23, 24].

The radiologist identified breast lesions and assigned a final assessment on a per-breast basis using a 1 to 5 scale that parallels BI-RADS assessment categories. Assessments were as follows: 1 (negative), 2 (benign), 3 (probably benign), and 4 (suspicious) or 5 (highly suggestive of malignancy) [23]. Assessments of 3 or higher were considered positive. Lesion size was measured from the CC or MLO views that best demonstrated the full extent of disease. The radiologist also gave an overall assessment of image quality on a 4-category scale (poor, suboptimal, acceptable, good).

### Statistical analysis

The proportions of malignant tumors and benign lesions detected by ^99m^Tc maraciclatide and ^99m^Tc sestamibi were compared using McNemar’s test for correlated proportions. A Wilcoxon signed-rank test was used to determine whether assessment scores differed between the ^99m^Tc maraciclatide and ^99m^Tc sestamibi studies.

Tumor extent measured on ^99m^Tc maraciclatide and ^99m^Tc sestamibi imaging was compared using a paired *t* test. The correlations between tumor extent measured on imaging and lesion extent measured at pathology were determined by Pearson correlation coefficient (r).

L/B ratios of lesions detected on ^99m^Tc maraciclatide and ^99m^Tc sestamibi imaging were compared using paired t-tests. A student’s *t*-test was used to compare L/B ratio between malignant and benign lesions for each radiopharmaceutical. Correlation in L/B ratio measured on ^99m^Tc maraciclatide and ^99m^Tc sestamibi MBI were also determined by Pearson correlation coefficient (*r*). Area under the curve (AUC), obtained from ROC analysis of both radiopharmaceuticals, was used to determine the value of the L/B ratio in discriminating between malignant and benign lesions. Correlation of L/B ratios with tumor subtype, considered as three ordinal categories (luminal, HER-2 enriched, triple negative), was determined by Spearman correlation coefficient (*r*_*s*_).

Statistical analyses were two sided with a significance level of 0.05.

## Results

A total of 40 patients were enrolled in this study. One patient withdrew after the initial MBI scan; thus, 39 patients successfully completed both MBI studies. In the analyzable 39 patients, average age was 56.5 years (SD 12.3, range 41–82). There were no reported adverse reactions.

Image quality was rated as good in all 39 ^99m^Tc maraciclatide studies. In the 39 ^99m^Tc sestamibi studies, 32 (82%) were rated as having good image quality and 7 (18%) were ranked as having acceptable image quality. Review of the lesion and background counts recorded from the images in patients who underwent the 1-day protocol, showed a ^99m^Tc maraciclatide/^99m^Tc sestamibi ratio of 2.4:1 in lesions and 2.3:1 in background. These ratios are slightly lower than the anticipated ratio of 2.7:1.

### Malignant lesions

**Fig. 1:**
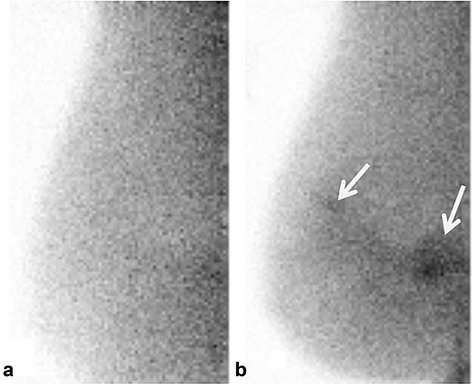
Molecular breast images in the mediolateral oblique projection from a 73-year-old patient with biopsy-proven invasive lobular carcinoma (Table 1, tumor #21). At blinded review, MBI performed with 300 MBq ^99m^Tc sestamibi (**a**) was interpreted as negative; MBI performed 3 days later with 300 MBq ^99m^Tc maraciclatide (**b**) was interpreted as assessment category 4. The lesion extent was 9.3 cm (*arrows*). Final pathology revealed grade I invasive lobular carcinoma of luminal A subtype, forming a 2.5-cm mass

A total of 21 patients had a diagnosis of breast cancer with a total of 21 malignant lesions identified (Table 1). Eighteen of 21 tumors (86%) were seen on ^99m^Tc sestamibi MBI and 19 of 21 (90%) were seen on ^99m^Tc maraciclatide MBI (*p* = 1). Eighteen tumors were seen with both radiopharmaceuticals, 1 was seen only on ^99m^Tc maraciclatide MBI, and 2 were not detected by MBI with either radiopharmaceutical. The tumor detected only by ^99m^Tc maraciclatide was an invasive lobular carcinoma that was 2.5 cm on pathology but had an apparent extent of 9.3 cm on maraciclatide MBI (Table 1, tumor #21; Fig. 1). The two malignant tumors not seen with either radiotracer included a 6-mm and a 7-mm invasive ductal carcinoma (Table 1, tumors #2 and #3).

**Table 1:** Summary of 21 cancers identified in 21 patients

Tumor number	Histopathology	Pathologic size (cm)	Molecular subtype	Assessment		Tumor extent (cm)		L/B ratio	
				^99m^Tc sestamibi	^99m^Tc maraciclatide	^99m^Tc sestamibi	^99m^Tc maraciclatide	^99m^Tc sestamibi	^99m^Tc maraciclatide
1	DCIS	7.0	Not applicable	4	4	10.3	8.3	1.54	1.79
2	IDC	0.6	Luminal	1	1	N/A	N/A	N/A	N/A
3	IDC	0.7	Luminal	1	2	N/A	N/A	1.02	1.15
4	IDC	0.8	Triple negative	3	4	1.2	1.1	1.16	1.15
5	IDC	0.9	Luminal	4	4	1.1	1.3	1.29	1.37
6	IDC	1.2	Luminal	4	4	3.1	3.1	1.59	1.71
7	IDC	1.5	HER-2 positive	4	5	1.5	1.3	1.62	1.60
8	IDC	1.6	HER-2 positive	5	4	2.1	1.7	1.93	2.70
9	IDC	1.7	Luminal	4	4	1.0	1.8	1.12	1.11
10	IDC	1.8	Triple negative	4	5	1.7	1.3	1.71	1.67
11	IDC	2.6	HER-2 positive	4	4	3.0	3.0	2.02	1.62
12	IDC	3.5	HER-2 positive	4	4	7.0	7.5	1.27	1.30
13	IDC	5.2	Luminal	4	4	3.0	3.3	1.57	1.50
14	IDC	5.4	Luminal	4	4	5.9	2.7	1.31	1.61
15	IDC	7.0	Luminal	5	4	3.0	3.0	1.63	1.79
16	IDC	9.2	Triple negative	4	4	11.0	9.2	1.43	1.42
17	Mixed IDC/ILC	1.1	Luminal	4	4	1.0	1.1	1.41	1.55
18	Mixed IDC/ILC	1.6	Luminal	4	4	6.5	6.3	1.68	1.68
19	Mixed IDC/ILC	3.7	Luminal	4	4	2.8	5.4	2.17	2.07
20	Mixed IDC/ILC	13.1	Luminal	5	5	11.4	12.5	2.31	2.09
21	ILC	2.5	Luminal	1	4	N/A	9.3	1.15	1.49

*DCIS* ductal carcinoma in situ, *IDC* invasive ductal carcinoma, *ILC* invasive lobular carcinoma in situ

**Fig. 2:**
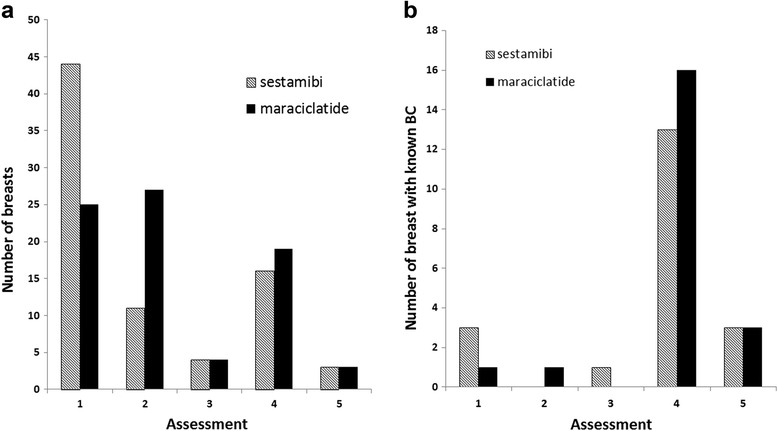
Distribution of assessment scores in **a** all 78 breasts and **b** 21 breasts with proven breast cancer

The overall distribution of assessment scores differed between ^99m^Tc sestamibi and ^99m^Tc maraciclatide (Fig. 2a; *p* < 0.001). However, this difference appears to be primarily due to the higher number of benign assessments (category 2) seen with ^99m^Tc maraciclatide relative to ^99m^Tc sestamibi and the corresponding lower number of negative assessments (category 1) seen with ^99m^Tc maraciclatide, as no difference was observed in assessment scores in the subset of patients with breast cancer (Fig. 2b; *p* = N.S.).

**Fig. 3:**
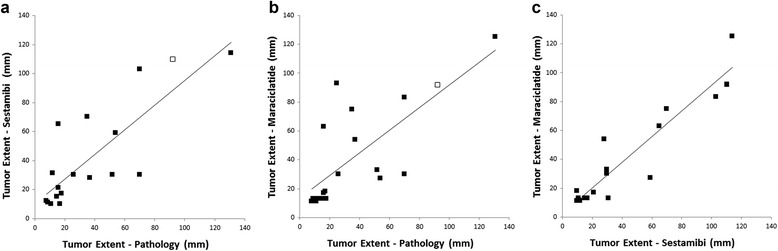
**a** Correlation between largest tumor extent recorded from pathology and largest tumor extent measured from ^99m^Tc sestamibi images in 18 malignant tumors seen on MBI (*r* = 0.82). Open square = neoadjuvant chemotherapy, size estimated from MRI. **b** Correlation between largest tumor extent recorded from pathology and largest tumor extent measured from ^99m^Tc maraciclatide images in 19 malignant tumors seen on MBI (*r* = 0.74). Open square = neoadjuvant chemotherapy, size estimated from MRI. **c** Correlation between tumor extent measured from ^99m^Tc sestamibi images and from ^99m^Tc maraciclatide images in 18 malignant tumors detected on both scans (*r* = 0.93)

**Fig. 4:**
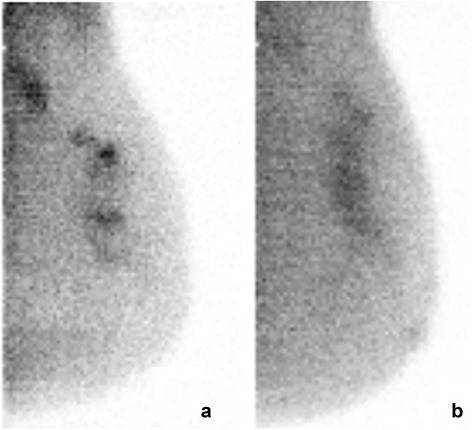
Molecular breast images in the mediolateral oblique projection from a 53-year-old patient with a palpable lesion that was suspicious on diagnostic mammography and scheduled for biopsy (Table 1, tumor #16). At blinded review, MBI performed with 300 MBq ^99m^Tc sestamibi (**a**) was interpreted as having moderate intensity radiotracer uptake in a segmental distribution with maximum extent of 11.0 cm. An assessment of 4 was assigned. MBI performed 1 day later with 300 MBq ^99m^Tc maraciclatide (**b**) was also interpreted as assessment 4 with lesion extent measuring 9.2 cm. Pathology revealed grade III invasive ductal carcinoma. Patient underwent subsequent neoadjuvant chemotherapy. Tumor extent measured on contrast-enhanced MRI prior to neoadjuvant chemotherapy was estimated at 9.2 cm

Average tumor size on pathology was 3.5 cm (SD 3.3 cm; range 0.6 to 13.1 cm). Tumor extent measured on MBI was similar for the two radiopharmaceuticals; average tumor size was 4.3 cm (SD 3.6 cm) on the ^99m^Tc sestamibi images and 4.3 cm (SD 3.5 cm) on the ^99m^Tc maraciclatide images (*p* = 0.62). Tumor extent measurements strongly correlated with pathology size (*r* = 0.82 for ^99m^Tc sestamibi and *r* = 0.74 for ^99m^Tc maraciclatide; Fig. 3a, b). Figure 3c shows the correlation between tumor extent measured from the ^99m^Tc sestamibi and ^99m^Tc maraciclatide images (*r* = 0.93). In one patient who underwent neoadjuvant chemotherapy (Fig. 4), no pathologic estimate of tumor size was available. Tumor extent from contrast-enhanced breast MRI performed prior to chemotherapy was used as a reference standard in this case.

### Benign lesions

**Fig. 5:**
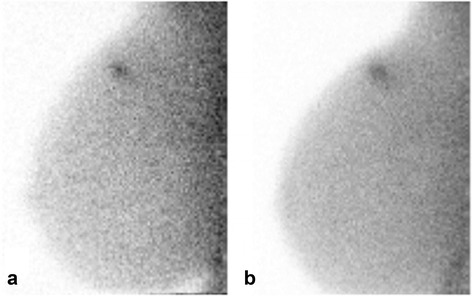
Molecular breast images in the mediolateral oblique projection from a 41-year-old patient with a palpable lesion that was suspicious on diagnostic mammography and scheduled for biopsy (Table 2, benign lesion #16). At blinded review, MBI performed with 300 MBq ^99m^Tc sestamibi (**a**) was interpreted as having a focal area of moderate intensity radiotracer uptake with maximum extent of 1.3 cm. An assessment of 4 was assigned. MBI performed the same day with 740 MBq ^99m^Tc maraciclatide (**b**) was also interpreted as assessment 4 with lesion extent measuring 1.6 cm. Biopsy revealed necrotizing granulomatous inflammation and fibrosis

Benign biopsy findings were found in 20 patients (Table 2). Nine of 20 (45%) benign lesions were seen on ^99m^Tc sestamibi MBI and on ^99m^Tc maraciclatide MBI. Eight benign lesions were seen with both radiopharmaceuticals, 1 atypical ductal hyperplasia was seen only with ^99m^Tc maraciclatide, and 1 intramammary lymph node was seen only with ^99m^Tc sestamibi. Ten were not detected by MBI with either radiopharmaceutical. Figure 5 shows an example of the relative findings on MBI with both ^99m^Tc sestamibi and ^99m^Tc maraciclatide in a patient with necrotizing granulomatous inflammation.

**Table 2:** Summary of 20 benign lesions in 20 patients

Benign lesion number	Histopathology	L/B ratio		Assessment	
		^99m^Tc sestamibi	^99m^Tc maraciclatide	^99m^Tc sestamibi	^99m^Tc maraciclatide
1	ADH	1.24	1.28	3	3
2	ADH^a^	Not seen	1.34	1	4
3	Benign fibrocystic changes	Not seen	Not seen	1	2
4	Benign fibrocystic changes	Not seen	Not seen	1	1
5	Benign fibrocystic changes	Not seen	Not seen	1	2
6	Benign fibrocystic changes	Not seen	Not seen	1	1
7	Benign fibrocystic changes	Not seen	Not seen	1	2
8	Benign fibrocystic changes	Not seen	Not seen	1	1
9	Fibroadenoma	1.52	1.35	3	3
10	Fibroadenoma	Not seen	Not seen	1	2
11	Fibroadenoma	Not seen	Not seen	1	1
12	Fibroadenoma	Not seen	Not seen	1	2
13	Fibroadenoma	1.24	1.13	3	4
14	Fibroadenoma	1.14	1.25	2	3
15	Fibroadenoma^b^	1.12	1.11	4	4
16	Inflammatory tissue	1.25	1.36	4	4
17	Intramammary lymph node	1.14	Not seen	4	2
18	Papilloma	Not seen	Not seen	2	2
19	Papilloma	1.07	1.13	2	2
20	PASH	2.35	2.76	2	2

*ADH* atypical ductal hyperplasia, *PASH* pseudoangiomatous stromal hyperplasia

^a^Patient with invasive ductal carcinoma had ADH diagnosed in the contralateral breast

^b^Patient with invasive ductal carcinoma had a fibroadenoma in the ipsilateral breast

### L/B ratio

**Fig. 6:**
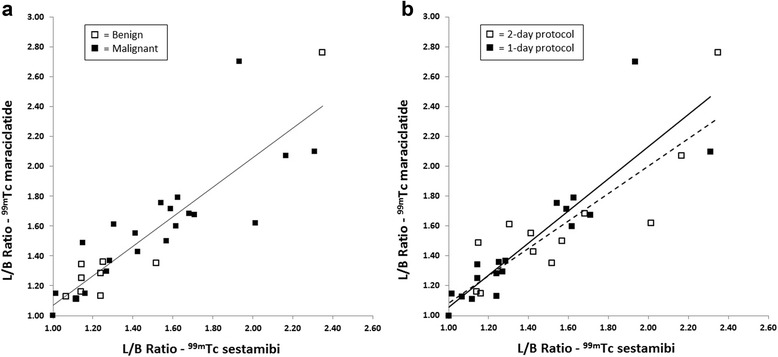
**a** Correlation between L/B ratio measured on malignant and benign lesions from ^99m^Tc sestamibi and ^99m^Tc maraciclatide images (malignant, *r* = 0.82; benign, *r* = 0.96; all lesions, *r* = 0.87). **b** Correlation between L/B ratio in malignant and benign lesions from ^99m^Tc sestamibi and ^99m^Tc maraciclatide images measured using the 1-day and 2-day protocols (1-day protocol, *r* = 0.89, *solid line*; 2-day protocol, *r* = 0.85, *dotted line*)

The L/B ratio in detected breast cancers was similar for the two radiopharmaceuticals: 1.55 ± 0.36 (mean ± SD) for ^99m^Tc sestamibi and 1.62 ± 0.37 (mean ± SD) for ^99m^Tc maraciclatide (*p* = 0.53) and strongly correlated (Fig. 6a; *r* = 0.82). The average L/B ratio for benign lesions was also similar for the two radiopharmaceuticals: 1.34 (SD 0.4) for ^99m^Tc sestamibi and 1.41 (S.D. 0.52) for ^99m^Tc maraciclatide (*p* = 0.75). The average L/B ratio for malignant lesions was higher than that for benign lesions for both ^99m^Tc sestamibi, (1.55 vs 1.34, *p* = 0.18.) and ^99m^Tc maraciclatide (1.62 vs. 1.41, *p* = 0.23), but the difference did not reach significance.

Figure 6b illustrates the correlation between lesion uptake (both malignant and benign) for ^99m^Tc sestamibi and ^99m^Tc maraciclatide as a function of the acquisition protocol. No apparent bias in terms of L/B ratio as a function of the type of protocol used to acquire the studies was observed.

**Fig. 7:**
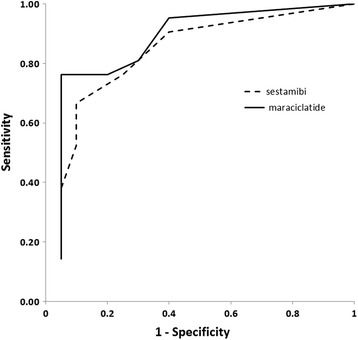
ROC analysis of L/B ratio comparing the sensitivity and specificity of ^99m^Tc sestamibi and ^99m^Tc maraciclatide images. The AUC was 0.83 and 0.87 for ^99m^Tc sestamibi and ^99m^Tc maraciclatide respectively (*p* = 0.64)

Figure 7 shows the results of the ROC analysis for ^99m^Tc sestamibi and ^99m^Tc maraciclatide. The overall diagnostic performance did not differ between the two radiopharmaceuticals. The AUC was 0.83 for ^99m^Tc sestamibi and 0.87 for ^99m^Tc maraciclatide (*p* = 0.64). A cut-off value of L/B = 1.2 yielded a sensitivity of 76% and a specificity of 75% for ^99m^Tc sestamibi and a sensitivity of 81% and a specificity of 70% for ^99m^Tc maraciclatide.

**Fig. 8:**
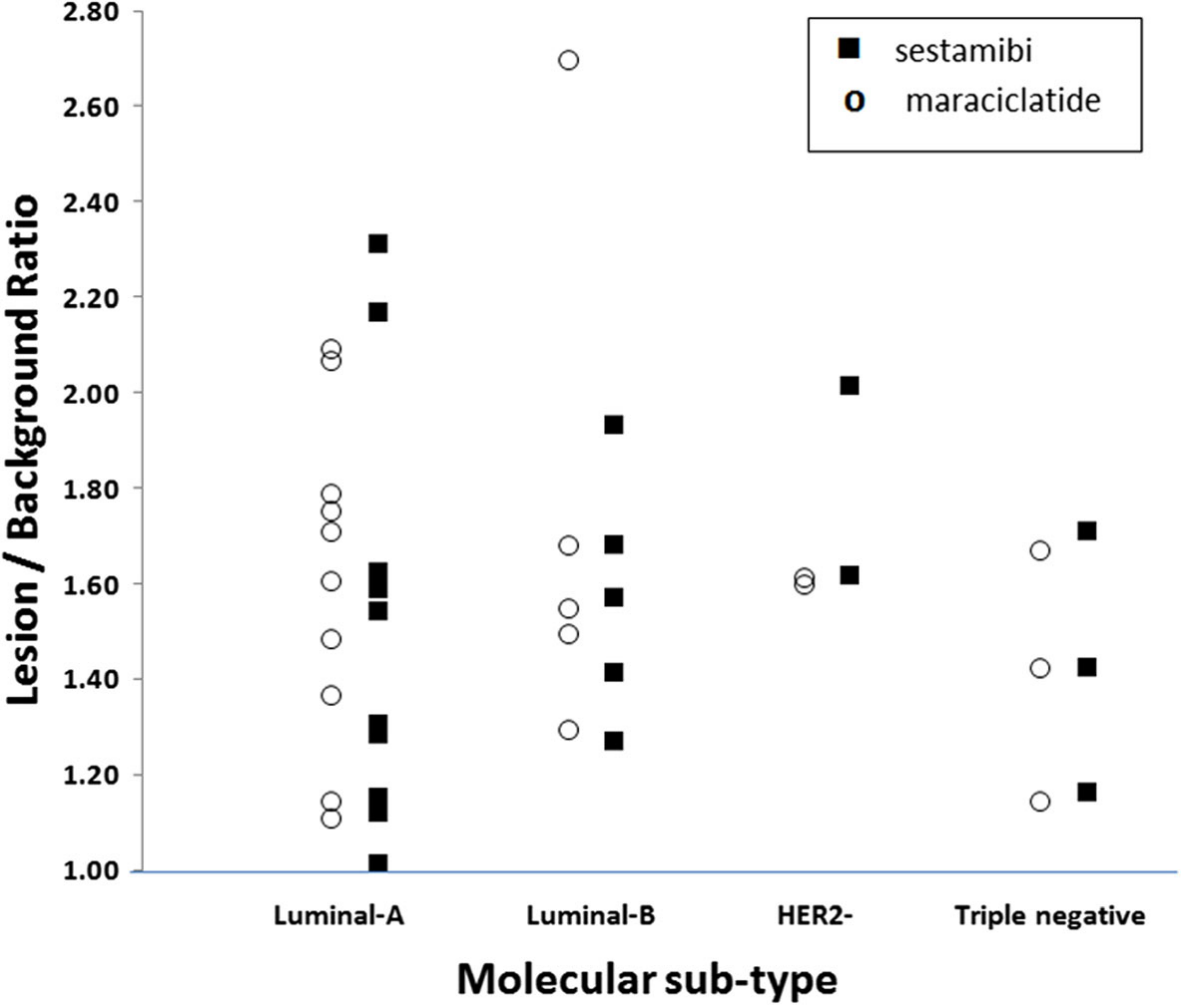
Distribution of L/B ratios by molecular subtype in the 20 known invasive cancers for ^99m^Tc sestamibi (*r*_*s*_ = 0.12, *p* = 0.63) and ^99m^Tc maraciclatide (*r*_*s*_ = −0.12, *p* = 0.64)

Figure 8 shows the distribution of the lesion to background (L/B) ratios measured from the ^99m^Tc sestamibi and ^99m^Tc maraciclatide images as a function of molecular subtype. No correlation was found between the L/B ratio and molecular subtype for ^99m^Tc sestamibi (*r*_*s*_ = 0.12, *p* = 0.63) or ^99m^Tc maraciclatide (*r*_*s*_ = −0.12, *p* = 0.64).

## Discussion

The overall results of this study show that ^99m^Tc maraciclatide offers comparable uptake in breast tumors to ^99m^Tc sestamibi. Lesion extent and L/B ratio measured from the ^99m^Tc maraciclatide images correlated closely with values measured from the ^99m^Tc sestamibi images. Our findings suggest that ^99m^Tc maraciclatide offers only a marginal improvement in sensitivity over ^99m^Tc sestamibi that did not reach significance in this study.

Both radiopharmaceuticals demonstrated comparable uptake in benign lesions. It was hoped that the degree of uptake of the ^99m^Tc maraciclatide in these lesions would be less than that of ^99m^Tc sestamibi, thereby providing better discrimination between benign and malignant lesions. However, factors that contribute to the uptake of ^99m^Tc sestamibi in some benign lesions appear to equally influence the uptake of ^99m^Tc maraciclatide. Previous studies have shown that angiogenesis in breast tissue is initiated at the start of hyperplasia before there is any morphological evidence of malignancy [25]. Hence, it is not unexpected that there would be uptake of ^99m^Tc maraciclatide in benign lesions. The uptake is similar to that observed with ^99m^Tc sestamibi, thereby offering little or no improvement in discriminating power between benign and malignant lesions. ROC analysis (Fig. 7) showed only a marginal, and non-significant, difference in the ability of the two radiopharmaceuticals to distinguish benign from malignant lesions.

Bach-Gansmo et al. [15, 16] were the first to report on the high sensitivity of ^99m^Tc maraciclatide for the detection of breast cancer. In their studies, no comparison of the relative uptake of ^99m^Tc maraciclatide with ^99m^Tc sestamibi was performed. Ma et al showed that an analog of maraciclatide, ^99m^Tc RGD, demonstrated marginally better uptake in breast cancers than ^99m^Tc sestamibi, but that difference was not statistically significant. Furthermore they found that ^99m^Tc RGD did not provide any significant advantage over ^99m^Tc sestamibi in distinguishing benign from malignant lesions [6]. In agreement with the findings of Ma et al, we found a good correlation between the uptakes of the 2 radiopharmaceuticals and no difference in the ability of either radiopharmaceutical to distinguish malignant from benign lesions (Fig. 7). Likewise, apparent lesion extent appeared to be similar with both radiopharmaceuticals.

^99m^Tc sestamibi is currently the only radiopharmaceutical that is FDA-approved for breast imaging [26]. Several recent studies have shown its clinical use as an imaging agent for the detection of breast cancer in the screening environment [27–29]. ^99m^Tc sestamibi also offers a practical advantage over ^99m^Tc maraciclatide in that it does not require a 45-min wait period post injection. With an excellent safety record and no serious adverse events associated with over 30 years of clinical use [30], ^99m^Tc sestamibi has essentially become the primary radiotracer used in the majority of MBI studies. Uptake of ^99m^Tc sestamibi in tumors is primarily dependent on blood flow to the tumor bed and on increased uptake in the mitochondria of the tumor cells [31]. As such, it has served as a general-purpose imaging agent for the detection of breast cancer, but these same characteristics also result in uptake in benign lesions and conditions such as fibroadenomata, papillomas, areas of inflammation, necrosis, and benign fibrocystic changes [32, 33].

^99m^Tc maraciclatide may offer some advantages over ^99m^Tc sestamibi for breast imaging. ^99m^Tc sestamibi is known to have significantly lower uptake in invasive lobular carcinomas than in invasive ductal carcinomas [8]. Figure 1 would indicate that ^99m^Tc maraciclatide may be a better imaging agent than ^99m^Tc sestamibi for the detection of invasive lobular carcinomas. However, the same figure shows that ^99m^Tc maraciclatide can occasionally overestimate disease. The reason for this overestimation is unknown but may reflect hyperplasia in the breast tissue surrounding the lesion. ^99m^Tc maraciclatide is extracted primarily through the hepatobiliary system with no uptake in the myocardium. In some patients, this may result in better breast image quality as the absence of myocardial activity adjacent to the breast may result in less scatter in the breast images. This point is well demonstrated in Fig. 5 where increased activity close to the chest wall is evident on the ^99m^Tc sestamibi image but absent from the ^99m^Tc maraciclatide image. Both radiopharmaceuticals have a comparable radiation dosimetry profile. The effective radiation dose from ^99m^Tc maraciclatide is 7.8 uSv/MBq [34] which is comparable to that from ^99m^Tc sestamibi (7.1 uSv/MBq) [35]. However, no efforts have been expended to determine if the uptake of ^99m^Tc maraciclatide in breast tissue could be modulated through patient preparation as has been done with ^99m^Tc sestamibi [22].

One potential role for breast imaging with MBI is in patients undergoing neoadjuvant chemotherapy (NAC) where early prediction of response to NAC offers a potential opportunity to change treatment strategy in cases of inadequate response. Previous studies have shown that quantitative assessment of tumor uptake of ^99m^Tc sestamibi demonstrated the ability to differentiate between pathological responders and non-responders as early as 3–5 weeks after initiation of NAC [36]. To date, there is little understanding of what these changes in tumor uptake represent. Our findings did not support an association between the uptake of either radiopharmaceutical and molecular subtype. While the Luminal cancers had some of the lowest L/B ratios, there was no clear relationship between uptake and molecular subtype. Additional studies will be needed to determine what relationship, if any, exists between the degree of uptake of either radiopharmaceutical in a tumor and molecular subtype or tumor histopathology.

This study had some limitations. The sample size of 40 patients limits our ability to detect small differences in the sensitivity of the two radiopharmaceuticals. A second limitation was the use of a 1-day protocol in approximately half of patients, imposed by limitations in patient’s availability and inability to return for a 2-day protocol. Figure 6b showed no evidence that L/B ratios measured using the 1-day protocol were biased high or low relative to L/B ratios measured using the 2-day protocol. However, in those patients who underwent the 1-day protocol, image quality with the ^99m^Tc maraciclatide images may have been better than that observed in the ^99m^Tc sestamibi images due to the increased administered dose and corresponding reduced noise in the clinical images. All of the MBI examinations acquired for this study were considered to be of adequate image quality for diagnostic interpretation and appropriate adjustments to image counts were made for ROI analyses. The order of MBI examinations with the two radiopharmaceuticals was not randomized due to restrictions with preparation of the ^99m^Tc maraciclatide; however, MBI studies were reviewed in a randomized order with the radiologist blinded to radiopharmaceutical and all clinical information in order to avoid biasing the interpretation.

## Conclusions

^99m^Tc maraciclatide was found to offer comparable lesion uptake to ^99m^Tc sestamibi, in both malignant and benign lesions. Lesion extent and uptake measured from the ^99m^Tc maraciclatide images correlated closely with values measured from the ^99m^Tc sestamibi images. Our findings suggest that ^99m^Tc maraciclatide offers only a marginal improvement in sensitivity over ^99m^Tc sestamibi that did not reach significance. Our findings did not support an association between the uptake of either radiopharmaceutical and molecular subtype of tumors.
